# Urinary biomarkers of PAHs, VOCs, OPEs, and metal(loid)s in firefighters responding in the wildland urban interface (WUI)

**DOI:** 10.1016/j.ijheh.2026.114877

**Published:** 2026-07-24

**Authors:** Miriam M. Calkins, Alexander C. Mayer, Derek J. Urwin, Steve Bertke, Christine Toennis, Shawn C. Beitel, Jeff Hughes, Jamie Gabriel, Antonia M. Calafat, Christopher M. Reese, Julianne Cook Botelho, Maria Ospina, Cynthia D. Ward-Momon, Jeffery M. Jarrett, Alberto J. Caban-Martinez, Judith M. Graber, Deborah Sammons, Kathleen M. DuBose, Jefferey L. Burgess

**Affiliations:** a National Institute of Occupational Safety and Health, Centers for Disease Control and Prevention, Cincinnati, OH, USA; b Los Angeles County Fire Department, USA; c Department of Chemistry & Biochemistry, University of California Los Angeles, Los Angeles, CA, USA; d International Association of Fire Fighters, Washington, D.C, USA; e Zuckerman College of Public Health, University of Arizona, Tucson, AZ, USA; f Orange County Fire Authority, USA; g Division of Laboratory Sciences, National Center for Environmental Health, Centers for Disease Control and Prevention, Atlanta, GA, USA; h Leonard M. Miller School of Medicine, University of Miami, Miami, FL, USA; i Rutgers, the State University of New Jersey, Piscataway, MJ, USA; j Office of Wildland Fire, Department of the Interior, USA; k Duke University, Division of Environmental Natural Sciences, Durham, NC, USA

**Keywords:** Firefighter, Wildland-urban interface, WUI, PAHs, OPEs, VOCs, Metals

## Abstract

Firefighters suppressing wildland urban interface (WUI) fires may be exposed to smoke from burning vegetation as well as structures and vehicles. To better understand firefighters’ internal dose exposures in the WUI environment, 250 firefighters from Southern California were enrolled into the Fire Fighter Cancer Cohort Study (FFCCS) in 2019, providing urine samples at enrollment and post-fire. One-hundred and twenty-five post-fire samples were collected from 89 participants. Samples were analyzed for metal(loid)s and metabolites of polycyclic aromatic hydrocarbons (PAHs), volatile organic compounds (VOCs), and organophosphate esters (OPEs). We assessed the difference between creatinine-corrected urine concentrations for each chemical at enrollment compared with post-fire, controlling for the individual participant. We also compared these concentrations to the general U.S. population concentrations from the National Health and Nutrition Examination Survey (NHANES) and the American Conference of Governmental Industrial Hygienists Biological Exposure Indices (BEIs) when available. All seven PAH and 11 VOC metabolite concentrations increased significantly from enrollment to post-fire. Diphenyl phosphate, antimony, and cadmium also significantly increased from enrollment to post-fire. Maximum post-fire concentrations in firefighters also exceeded the BEI for 1-hydroxyprene and trans,trans-muconic acid (a metabolite of benzene). Firefighters assigned to structure defense had significantly higher increases in PAH metabolites compared to those assigned to wildland firefighting tasks. Personal protective equipment (PPE) usage was not evaluated in this study, but future studies could combine personal sampling with the collection of detailed PPE usage to develop effective practices and decontamination strategies during WUI fire responses.

## 1. Introduction

Occupational exposures experienced by firefighters are complex and highly heterogeneous. Fire response activities are diverse, influenced by characteristics of the response, geography, and work and employment structure. Fuels, job tasks, and use of personal protective equipment (PPE) are known to affect exposure to hazards that could influence the development of adverse health outcomes (Cherry et al., 2023; Fent et al., 2020; Navarro et al., 2023). Research from structural and wildland fire responses indicates that firefighters are exposed to numerous chemicals, including polycyclic aromatic hydrocarbons (PAHs), volatile organic compounds (VOCs), organophosphate esters (OPEs), and metal(loid)s (Engelsman et al., 2020; Mayer et al. 2021, 2023; Paiva et al., 2024).

Firefighters are increasingly responsible for responding to fires in geographic areas where human development (e.g. houses and other structures) intermingles with undeveloped wildland vegetation, often referred to as the wildland urban “interface” or “intermix” (WUI) environment (NIST, 2023). WUI fires tend to resemble wildfires in intensity and duration, and often require fire response strategies utilized for wildfire response. For example, unlike most structural responses which are contained within a matter of hours, WUI responses may involve prolonged periods (e.g. ≥ 8 h shifts over multiple days) of high levels of physical exertion, fire attack, and work in extreme heat; access by vehicle and water resources may be limited depending on the location of the fire and surrounding damage. While WUI fires routinely occur on smaller scales, their frequency and intensity around the globe are increasing. An illustrative example of one of the most destructive WUI fires in recent U.S. history, the 2018 Camp Fire in Butte County, CA, took 17 days to contain, burned 153,336 acres, damaged or destroyed 18,804 structures, and resulted in 85 fatalities (NASEM, 2022). In addition, the use of PPE recommended for urban structure fire responses, particularly respiratory protection (i.e. self-contained breathing apparatus), may be limited by available resources and the nature of response activities (Hwang et al., 2023).

The mixture of chemical hazards present in WUI fuels includes both vegetative material (Hwang et al., 2023) as well as materials typical of urban environments, including residential structures, industrial facilities, and vehicles (NIOSH, 2024). While research characterizing WUI exposures is limited, bounding estimates based on studies of urban and wildland environments indicate emissions of PAHs and other organic compounds from urban fuels may be substantial in WUI incidents (Holder et al., 2023). Evidence from post-WUI fire investigations demonstrates that these events may contribute to environmental and occupational metal(loid) exposure (NIOSH, 2019).

Additionally, WUI fires result in possible exposures to synthetic chemicals like OPEs when materials in homes are burned. Specifically, a recent evaluation found triphenyl phosphate (TPhP) in personal air samples and elevated post-fire diphenyl phosphate (a non-specific metabolite of TPhP) concentrations taken from firefighters responding to realistic training fire scenarios where the fuel load included furnishings with OPEs (Fent et al., 2020; Mayer et al., 2021). Among first responders for the Maui 2023 Wildfires, firefighter urine contained elevated concentrations of some OPEs, compared to police officers, ocean safety officers, and other county employees (NIOSH, 2024).

Occupational exposure as a firefighter was designated carcinogenic to humans (Group 1) by the International Agency for Research on Cancer (IARC) in 2022, based on sufficient evidence for bladder cancer and mesothelioma (Demers et al., 2022). Exposure to PAHs, VOCs, OPEs, and metal(loid)s are associated with adverse health effects, including neurotoxicity, developmental toxicity, nephrotoxicity, hepatotoxicity, cardiovascular toxicity, and cancer. More specifically, TPhP has been shown to be toxic to human cells (An et al., 2016), and several PAHs, VOCs, and metal(loid)s have been identified as known (benzene, benzo (a)pyrene, 1-3-butadiene, arsenic, cadmium), probable (acrolein, styrene, lead), or possible (naphthalene, acrylonitrile, crotonaldehyde, molybdenum) carcinogens by IARC (IARC, 2012). In addition to diagnosed health outcomes, exposure to the fire environment has known epigenetic effects, including changes in miRNA expression, DNA methylation, and epigenetic aging (Esteves et al., 2025; Quaid et al., 2024). Recently, Goodrich et al., (2025) demonstrated effects on miRNA expression specifically associated with WUI fire response (Goodrich et al., 2025).

A recent review article assessing wildland and structural firefighting exposures identified the need to evaluate the distinct nature of exposures during WUI fires (Wah et al., 2025). Characterizing WUI fire exposures is particularly important as the WUI environment continues to expand and firefighters’ use of PPE during these responses is inconsistent (Hwang et al., 2023). To date, we are unaware of any large studies evaluating the internal dose of a wide range of exposures to hazardous chemicals during WUI fire responses. The aim of this study was to evaluate firefighter exposures to PAHs, OPEs, VOCs, and metal(loid)s through biomonitoring of urine collected at enrollment into the study and after responding to WUI fires.

## 2. Methods

### 2.1. Study design and population

In fall 2019, we enrolled firefighters in the Fire Fighter Cancer Cohort Study (FFCCS)—a national community-engaged prospective multicenter cohort study, from two county fire departments in southern California. We enrolled 250 firefighters to increase the likelihood that firefighters responding to WUI incidents would also be participants of this study. Enrollment, specimen collection, and survey administration followed the FFCCS protocol, described more completely elsewhere (Burgess et al., 2025). In brief, participants provided a spot enrollment urine sample and completed a questionnaire containing sections on demographics and work history at enrollment; additional spot urine samples were collected following WUI fire responses over an eight month period in 2019. Of the 250 enrolled participants, 89 provided a urine sample after responding to at least one WUI incident in 2019; 88 of these participants also provided a urine sample at enrollment. A total of 126 post-fire urine samples were collected; however, one individual was not able to be linked with their study ID, so 125 samples were included in the analysis. Rank and assignment were ascertained from the questionnaire and incident notes from researchers in the field. The ranks of firefighter and hand crew firefighter are distinct. In this study, firefighters (as well as driver operators and captains) refer to members trained and certified as structural firefighters who responded to WUI fires on Type I fire engines equipped with fire pumps and hose. These members may be assigned to structure defense, progressive hose lays, cutting line, etc. and are trained to respond to wildfires (wildland and WUI), in addition to their structural firefighting duties. Hand crew firefighters refer to members whose regular duties do not include responding to structure fires, who work with hand tools and chain saws to cut lines during wildfire responses, and typically are not equipped with a fire pump or hose. Rank was captured for all participants, but incident-specific information was only captured for a subset of the population.

This study was reviewed and approved by the institutional review board (IRB) of the University of Miami (IRB approval no. 20170997) and participating institutions established reliance out agreements (§ See 45 C.F.R. part 46.114; 21 C.F.R. part 56.114). The analysis of de-identified specimens at the Centers for Disease Control and Prevention (CDC) laboratory was determined not to constitute engagement in human subjects research.

### 2.2. Urine sampling and chemical analysis

Firefighters provided spot urine samples at enrollment into the study and within approximately one to 3 h following the end of their shift where WUI firefighting operations were performed. Participants were instructed to wash their hands and provide a urine sample in sterile 120-ml polypropylene specimen collection cups that were pre-screened for metal(loid)s. Samples were initially stored in a cooler on ice during transport. Due to field conditions and ongoing fire response, some samples were stored on dry ice or in a −20 C freezer prior to transportation from the field to the lab. For most samples, within 10 h of collection, a 3.0 mL aliquot was dispensed into a 5 mL polypropylene cryovial containing 30 μL of 200 g/L sulfamic acid, 0.01% Triton® X-100 as a preservative, for the measurement of urinary mercury. The samples and mercury aliquot were then frozen and shipped on dry ice to the University of Arizona to be stored at −80 °C pending analysis for exposure biomarkers and creatinine. To facilitate collection of samples from participants in remote response locations, local firefighter research champions with IRB/human subjects CITI or Community Involvement in Research Training (CIRT) certification and the ability to enter WUI fire zones assisted with coordination of sample collection during fire response (Anderson, 2015).

Urine samples were analyzed for standard panels of PAHs, VOCs, OPEs, and metal(loid)s (See Supplement Table 1) at CDC’s National Center for Environment Health laboratories. PAHs included seven metabolites in four PAH biomarker categories: hydroxynaphthalenes (1-hydroxynaphthalene (1-NAP) and 2-hydroxynaphthalene (2-NAP)), hydroxyfluorenes (2-hydroxyfluorene (2-FLU) and 3-hydroxyfluorene (3-FLU)), hydroxyphenanthrenes (1-hydroxyphenanthrene (1-PHE) and 2-3-hydroxyphenanthrene (2-3PHE)), and 1-hydroxypyrene (1-PYR). Urinary PAH metabolites were quantified using enzymatic hydrolysis followed by isotope dilution mass spectrometry (Wang et al., 2017). The limit of detection (LOD) ranged from 0.008 to 0.09 μg per liter (μg/L), depending on the PAH metabolite. Twenty-four VOC metabolites (Supplemental Table 1) were measured in urine by utilizing isotope dilution ultrahigh-performance liquid chromatography with electrospray tandem mass spectrometry. The LODs ranged from 0.150 to 29.5 μg/L for VOC metabolites.

The panel of urinary OPE biomarkers, measured following Jayatilaka et al., (2019) method (Jayatilaka et al., 2019) included 9 OPE metabolite biomarkers: DPhP, bis(1,3-dichloro-2-propyl) phosphate(BDCPP), bis (1-chloro-2-propyl) phosphate (BCPP), bis(2-chloroethyl) phosphate (BCEtP), dicresyl phosphates (DCP), dibutyl phosphate (DBuP), dibenzyl phosphate (DBzP), 2-((isopropyl)phenyl)phenyl phosphate (iPPPP), 4-((tert-butyl)phenyl)phenyl phosphate(tBPPP), and 2,3,4,5-tetrabromobenzoic acid (TBBA), a metabolite of the non-PBDE brominated flame retardant 2-ethylhexyl-2,3,4,5-tetrabromobenzoate. This method relies on enzymatic deconjugation of target metabolites followed by cleanup using automated off-line solid phase extraction and separation and detection by high-performance liquid chromatography and isotope dilution tandem mass spectrometry. Detection limits ranged from 0.05 to 0.5 μg/L, accuracy from 89 to 118%, and imprecision was <10%. Arsenic (total), chromium, and nickel were measured in urine by inductively coupled plasma universal cell mass spectrometry (ICP-UCT-MS); 17 other urine metal(loid)s were measured by ICP-UCT-MS and inductively coupled plasma dynamic reaction cell mass spectrometry (ICP-DRC-MS) (Caldwell et al., 2005; Jarrett et al., 2008; Jones et. 2021; CDC, 2018). The LODs ranged from 0.0024 to 3.2 μg/L for metal(loids). To adjust for dehydration, urine samples were measured for creatinine using a Vitros Autoanalyzer (Johnson & Johnson, New Brunswick, NJ) with a Vitros CREA slide by investigators at the National Institute for Occupational Safety and Health (NIOSH) at the CDC.

### 2.3. Statistical analysis

Summary statistics are presented as n (number of samples), n below the LOD, geometric mean (GM) and geometric standard division (GSD), median, and concentration range stratified by collection period (enrollment and post-fire). In calculating the descriptive statistics, concentrations below the LOD were assigned values by dividing the LOD by the square root of two (Hornung, R. W., & Reed, L. D., 1990). Creatinine-corrected urine concentrations were compared to concentrations for the general adult U.S. population reported in the National Health and Nutrition Examination Survey (NHANES), stratified by smoking status, when available.

Differences between enrollment and post-fire PAH, VOC, OPE, and metal(loid) concentrations were compared using linear mixed-effects models, with exposure period as a fixed effect and participant as a random intercept. The concentrations on the original scale did not meet normality assumptions, so biomarker concentrations were log-transformed, allowing the results to be interpreted as differences in geometric means (equivalent to medians on the original scale). To evaluate differences between enrollment and firefighters’ first post-fire concentrations by rank, a Kruskal-Wallis test was performed. Incident response information was captured from a subset of the population (74 participants). A Wilcoxon rank-sum test was used to evaluate differences between enrollment and firefighters’ first post-fire concentrations by their primary assignment. To visualize significant results from the Kruskal-Wallis and Wilcoxon rank-sum tests, box and whisker plots with minimum, 25th percentile, median, 75th percentile, and maximum were created. All statistical tests and visualizations were performed in R version 4.40 and were two-sided at the 0.05 significance level.

## 3. Results

### 3.1. Firefighter characteristics and summary of post-WUI change in urinary exposure biomarkers

With a mean age of 37.9 years, the majority of the 89 participating firefighters were male (97.8%), non-Hispanic (65%), and white (79%). Over 95% of firefighters reported being non-smokers. Just under half reported less than 10 years of experience as a firefighter (48%) (Table 1). The firefighter population represented a range of ranks, including hand crew firefighters (14%), firefighters (43%), driver operators (19%), captains (21%), and battalion chiefs (3%).

While all 89 participants provided post-WUI response urine samples from at least one incident, 36 samples were collected following additional WUI responses (at least 24 h after the previous sample collection) for a total of 125 post-WUI response urine samples (Table 2). Incident response characteristics were recorded for a subset of 103 post-WUI response samples from 74 participants. Types of fuel recorded included both vegetation and structures in 74% of the responses, and the majority of participants were assigned to structure defense (Table 2).

Creatinine-corrected urine concentrations were significantly higher (p-values <0.05) in post-fire samples compared with enrollment samples for 21 compounds and significantly lower for 4 compounds. Post-fire increases were observed for all PAH metabolites (36.4% - 429% median increase). Eleven VOC metabolites showed significant post-fire increases with median changes ranging from 11% to 796%. The VOC metabolites with significant increases included 2-methylhippuric acid [2MHA], a metabolite of xylene, N-Acetyl-S-(2-carbamoylethyl)-L-cysteine [2CaEMA], a metabolite of acrylamide, N-Acetyl-S-(2-carboxyethyl)-L-cysteine [2CoEMA], a metabolite of acrolein, N-Acetyl-S-(2-cyanoethyl)-L-cysteine [2CyEMA], a metabolite of acrylonitrile, N-Acetyl-S-(4-hydroxy-2-methyl-2-buten-1-yl)-L-cysteine [4HMBeMA], a metabolite of isoprene, N-Acetyl-S-(3,4-dihydroxybutyl)-L-cysteine [34HBMA] and N-Acetyl-S-(*4-hydroxy-2-buten-*1-yl)-L-cysteine [(E)-4HBeMA and (Z)-4HBeMA], both metabolites of 1,3 butadiene, N-Acetyl-S-(3-hydroxypropyl-1-methyl)-L-cysteine [3HMPMA], a metabolite of crotonaldehyde, mandelic acid [MADA], a metabolite of styrene and ethylbenzene, and *trans*, *trans*-muconic acid (MUCA) and N-Acetyl-S-(phenyl)-L-cysteine (*PhMA*), both metabolites of benzene.

One OPE metabolite, DPhP, showed a post-fire increase (116% increase). Medians for two metal(loid)s also increased: antimony (88% increase) and cadmium (13% increase). Post-fire decreases were observed for one VOC metabolite, N-Acetyl-S-(n-propyl)-L-cysteine (1PMA, a metabolite of 1-bromopropane: 29% decrease), and three metal(loid)s: barium (34% decrease), iodine (23% decrease), and thallium (14% decrease). Concentrations of biomarkers with no significant differences are reported in Supplement Tables 2–4.

### 3.2. Urinary PAH metabolite concentrations for firefighters responding to WUI fires

Most PAH metabolites were detected in 100% of enrollment and post-fire samples, except 3-FLU and 1-PYR (Table 3). Overall, the greatest difference was observed for 1-NAP, where the median concentration was 5.31 times higher post-fire (4.39 μg/g creatinine) than at enrollment (0.83 μg/g creatinine). Compared with the general population, enrollment and post-fire median concentrations of all PAH metabolites at enrollment were below the 95th percentile for non-smokers from 2015 to 2016 NHANES. Ten of the 125 post-fire samples had 1-PYR concentrations that exceeded the American Conference of Governmental Industrial Hygienists (ACGIH) Biological Exposure Indices (BEI®) of 2.5 μg/L (ACGIH, 2025).

### 3.3. Urinary VOC metabolite concentrations for firefighters responding to WUI fires

Eleven of the 24 VOC metabolites characterized in this study had significantly higher creatinine-corrected concentrations post-fire compared with enrollment: 2MHA, 2CaEMA, 2CoEMA, 2CyEMA, 34HBMA, 3HMPMA, 4HMBeMA, MADA, (E)-4HBeMA/(Z)-4HBeMA, MUCA, and PhMA (Table 4). Notably, median 2CyEMA concentrations were almost 9 times higher post-fire (8.69 μg/g creatinine) compared with enrollment (0.97 μg/g creatinine). Additionally, median concentrations of the metabolites of benzene doubled (PhMA = 92% higher; MUCA = 116% higher) from enrollment to post fire. While most maximum VOC metabolite concentrations were well below their respective ACGIH BEIs®, maximum MUCA concentrations at enrollment (3% of samples) and post-fire (5% of samples) exceeded the MUCA BEI of 500 μg/g creatinine. Maximum concentrations for PhMA were below the BEI of 25 μg/g creatinine. No significant differences were observed for all other urinary VOC metabolites (Supplemental Table 2).

### 3.4. Urinary OPE metabolite concentrations for firefighters responding to WUI fires

DPhP was the only urinary OPE metabolite with a significantly higher median creatinine-corrected concentration post-fire (1.79 μg/g creatinine) compared with enrollment (0.83 μg/g creatinine) (Table 5). Compared to the general population, concentrations of DPhP exceeded the 95th percentile of the 2017-2018 NHANES population at enrollment and post-fire in 5% and 20% of samples, respectively. No other significant differences were observed for urinary OPE metabolites (Supplemental Table 3).

### 3.5. Urinary metal(loid) concentrations for firefighters responding to WUI fires

Creatinine-corrected median concentrations for five of the twenty metal(loid)s measured in urine significantly differed post-fire compared with enrollment, with significantly lower concentrations observed for iodine, barium, and thallium and higher concentrations observed for antimony and cadmium post-fire (Table 6). The median concentration for antimony post-fire (0.081 μg/g creatinine) was nearly double that of enrollment (0.043 μg/g creatinine). Conversely, the median iodine concentration was 23% lower post-fire (57.82 μg/g creatinine) than at enrollment (75.41 μg/g creatinine). Although chromium was not detected in the majority of samples, 19 (8 at enrollment and 11 post-fire) samples exceeded the ACGIH BEI for chromium of 0.7 μg/L. No significant differences were observed for other metal(loid)s, and concentrations were generally unremarkable (Supplemental Table 4).

### 3.6. Impact of rank and primary incident job assignment on changes in urinary metabolites concentrations from enrollment to after the first WUI fire response

**Fig. 1.**
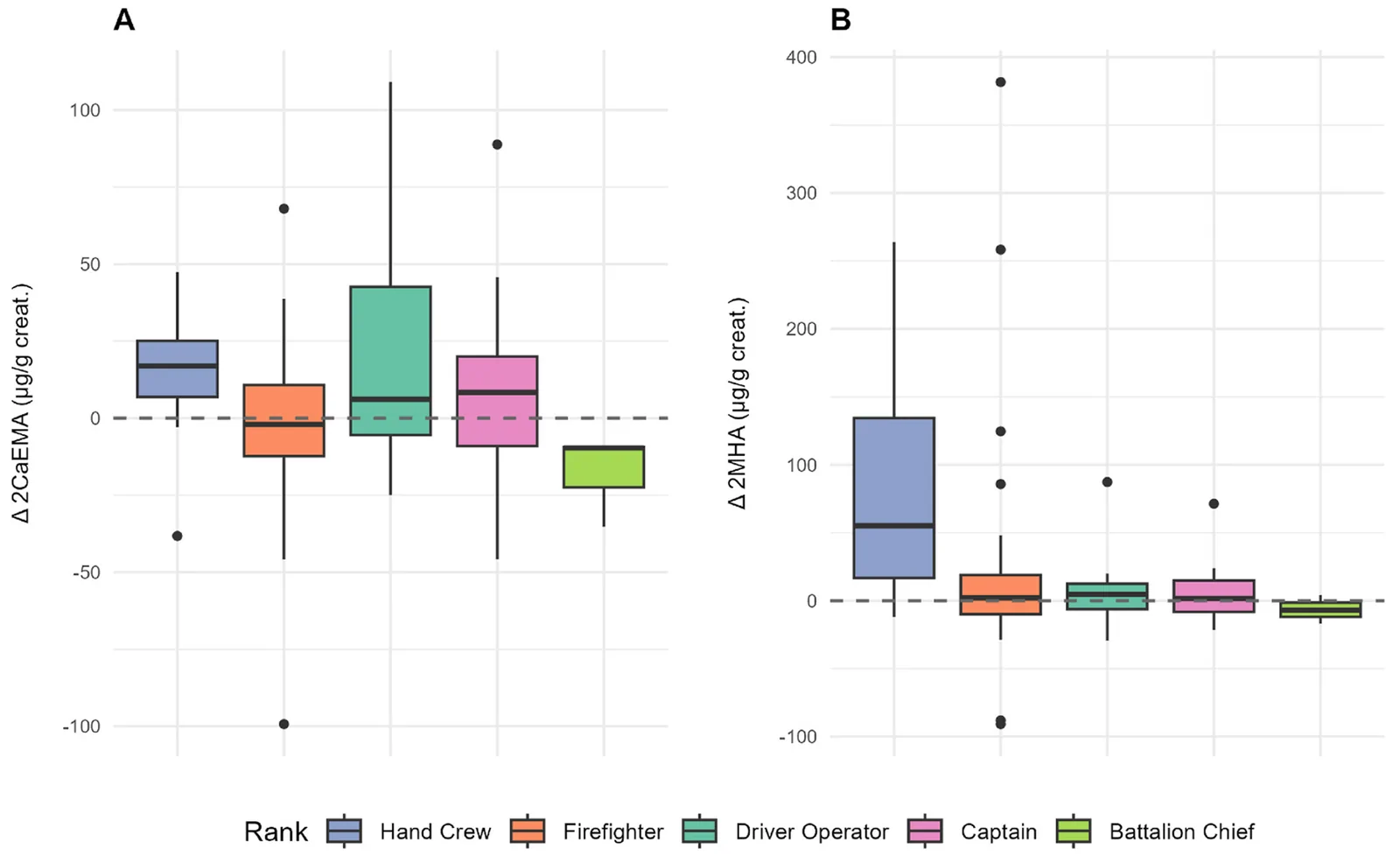
Change in creatinine-corrected urinary concentrations of (A) N-acetyl-S-(2-carbamoylethyl)-L-cysteine (2CaEMA, metabolite of acrylamide) and (B) 2-methylhippuric acid (2MHA, metabolite of xylenes) from enrollment to after their first WUI response, stratified by rank (Hand Crew-n = 11, Firefighter-n = 38, Driver Operator-n = 16, Captain-n = 19, Battalion Chief-n = 3). These represent the VOC metabolites where a significant difference by firefighting rank was observed (y-axis scales differ). The box represents the interquartile range (IQR) and the line in each box represents the median. The upper whisker represents the far upper fence 1.5 IQR above the 75th percentile, the lower whisker represents the lower fence 1.5 IQR below the 25th percentile, and the dots represent outliers.

**Fig. 2.**
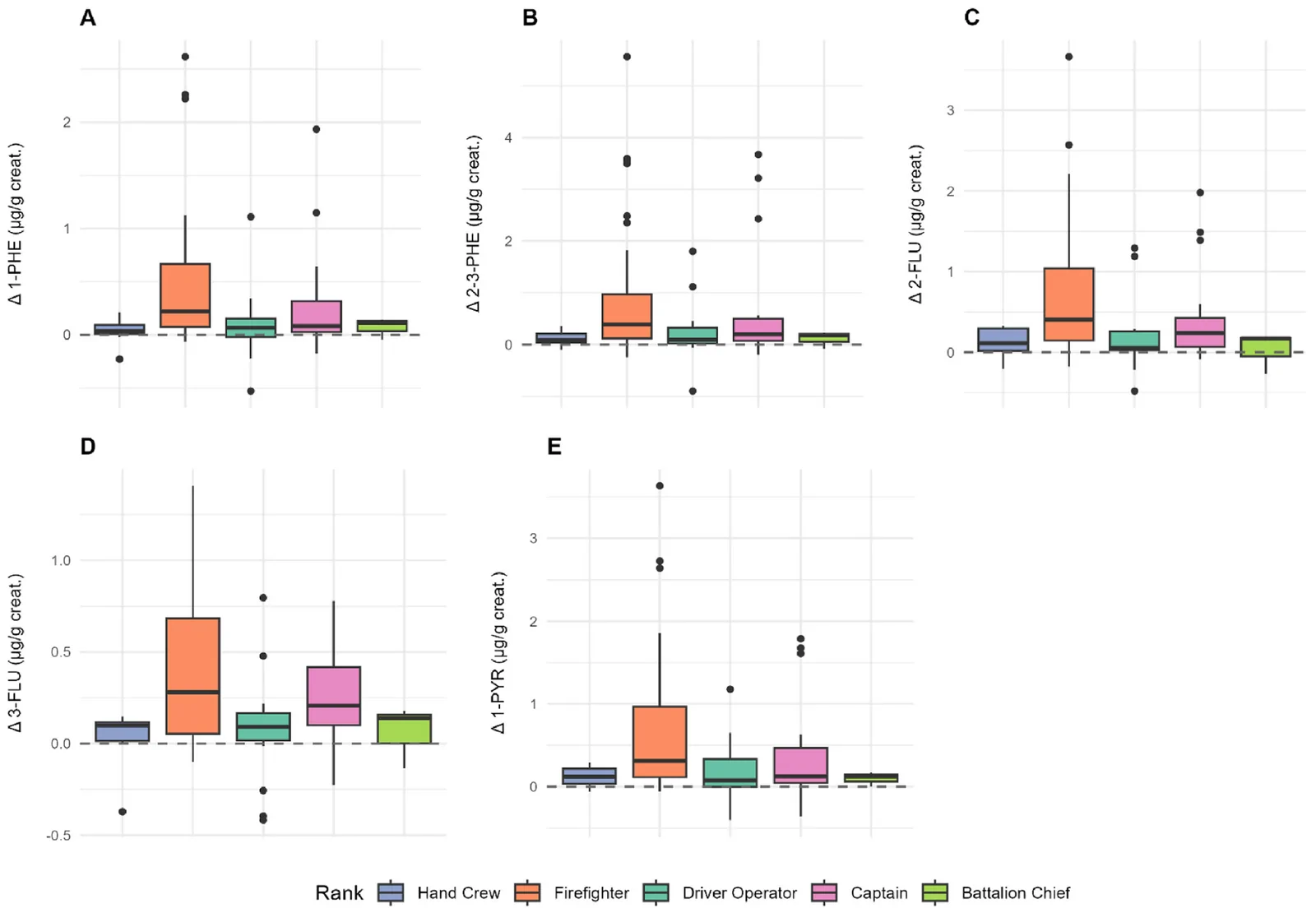
Change in creatinine-corrected urinary concentrations of (A) 1-hydroxyphenanthrene (1-PHE), (B) 2-3-hydroxyphenanthrene (2-3PHE), (C) 2-hydroxyfluorene (2-FLU), (D) 3-hydroxyfluorene (3-FLU), and (E) 1-hydroxyprene (1-PYR) from enrollment to after their first WUI response, stratified by firefighting rank (Hand Crew-n = 11, Firefighter-n = 38, Driver Operator-n = 16, Captain-n = 19, Battalion Chief-n = 3). These represent the PAH metabolites where a significant difference by rank was observed (y-axis scales differ). The box represents the interquartile range (IQR) and the line in each box represents the median. The upper whisker represents the far upper fence 1.5 IQR above the 75th percentile, the lower whisker represents the lower fence 1.5 IQR below the 25th percentile, and the dots represent outliers.

Fig. 1 presents the changes in creatinine-corrected urinary VOC metabolite concentrations from enrollment to post-first WUI response for the metabolites where a significant effect by firefighting rank was observed. Specifically, hand crew firefighters had the largest change in concentrations for both 2MHA (xylenes) and 2CaEMA (acrylamide) compared to the other ranks. Changes in urinary PAH metabolite concentrations where significant rank-based differences were observed are presented in Fig. 2. In general, firefighters (primarily structural) had larger increases in urinary PAH metabolites (2-FLU, 3-FLU, 1-PHE, 2-3PHE, and 1-PYR) from enrollment to post-fire response compared to other ranks.

**Fig. 3.**
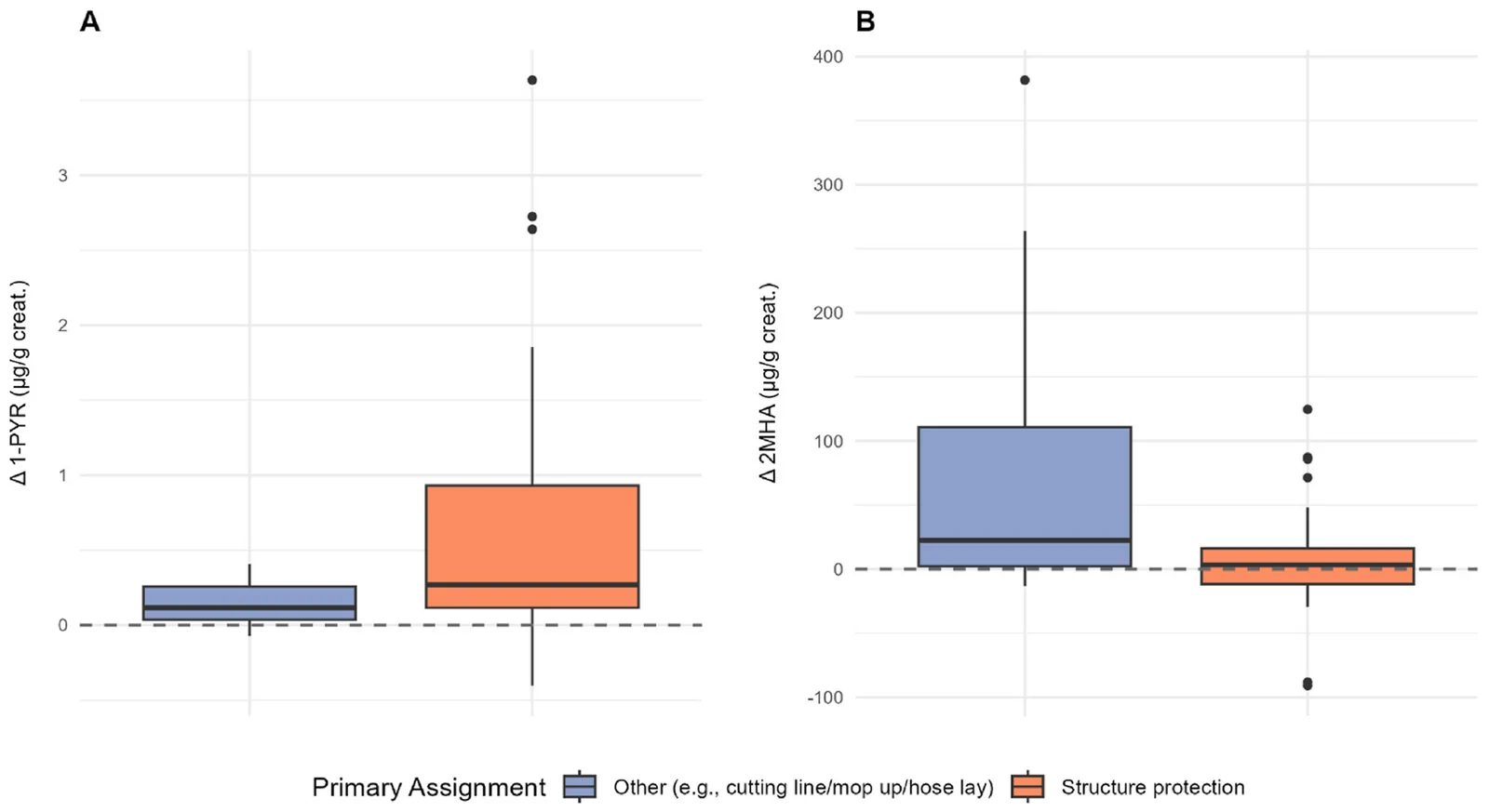
Change in creatinine-corrected urinary concentrations of (A) 1-hydroxypyrene (1-PYR) and (B) 2-methylhippuric acid (2MHA, metabolite of xylenes) from enrollment to after their first WUI response, stratified by primary assignment (Other-n = 14; Structure defense-n = 59). These represent the metabolites where a significant difference by primary assignment was observed (y-axis scales differ). The box represents the interquartile range (IQR) and the line in each box represents the median. The upper whisker represents the far upper fence 1.5 IQR above the 75th percentile, the lower whisker represents the lower fence 1.5 IQR below the 25th percentile, and the dots represent outliers.

Fire response information was collected from 74 participants during at least one WUI response. Among the metabolites that showed a significant difference from enrollment to post-fire, changes in 1-PYR and 2MHA concentrations varied significantly by assignment type (p-values <0.05; Fig. 3). Those assigned to structure defense had a change in 1-PYR concentrations that was significantly higher than those assigned to wildland firefighting tasks (i.e., other, e.g., cutting line, mop up, progressive hose lay), while the change in 2MHA was significantly higher in firefighters assigned to tasks associated with wildland firefighting compared to those assigned to structure defense.

## 4. Discussion

To our knowledge, this is one of the first studies to assess firefighters’ internal dose to a broad range of chemical hazards in the WUI fire environment. Fire incident response and geographic data from partner fire departments confirm that participants were engaged in fighting WUI fires in environments consisting of both vegetative and structural fuels. Thus, exposures reported here are likely a robust characterization of what firefighters may experience when responding to WUI fires in California. The observation of significantly higher post-fire creatinine-corrected median urinary concentrations for two metal(loid)s and biomarkers of all PAHs, multiple VOCs, and one OPE demonstrates that firefighters are exposed to chemical hazards during WUI fire responses. These findings underscore the value of biomonitoring for characterizing short-term internal dose associated with WUI fire response activities.

In this analysis, we observed a significant increase in all seven PAH metabolites from enrollment to post WUI fire responses, with median post-fire concentrations comparable to those reported after wildland fires (Adetona et al., 2017; Cherry et al., 2023; Mayer et al., 2025). Fent et al. (2020) evaluated PAH metabolites in structural firefighters conducting interior operations (i.e., fire attack and search and rescue) in a burning structure and reported post-fire median concentrations of hydroxyfluorenes, hydroxyphenanthrenes, and 1-hydroxypyrene that were higher than those found in the current study (Fent et al., 2020). A recent meta-analysis of post-fire urinary PAH metabolites in firefighters also found that those engaged in structural firefighting activities had significantly higher levels of PAH metabolites compared to those involved in wildland firefighting (Hwang et al., 2022). Notably, we also found that firefighters assigned to structure defense had significantly higher changes in 1-hydroxypyrene from enrollment to after the first WUI fire response. When we compared biomarker concentrations by rank, the rank of firefighter had significantly higher changes in PAH metabolites relative to hand crew firefighters, driver operators, captains, and battalion chiefs. Several post-fire urinary 1-hydroxpyrene concentrations for firefighters involved in structure defense in the current study were also above the peak concentrations previously found for firefighters assigned to interior operations during structure fire responses (Fent et al., 2020) and well above the ACIGH BEI of 2.5 μg/L (ACGIH, 2025). Overall, results from our study suggest that PAH exposure during WUI fire responses aligns more closely with exposures noted during wildland fire responses as opposed to structural firefighting. However, firefighters involved in structure defense during WUI fire responses can be exposed to levels of PAHs on par with what has been characterized from structural firefighters assigned to interior operations.

Both metabolites of benzene, MUCA and PhMA showed a significant increase from enrollment to post-fire among all firefighters. Median MUCA concentrations in our study rose to levels exceeding those previously reported for structural firefighters and fire instructors after realistic training fires (Fent et al., 2022). Several MUCA concentrations measured at enrollment and post-fire were also significantly above the ACGIH BEI of 500 μg/g creatinine (ACGIH, 2025). Given that preserved food containing sorbic acid can contribute to MUCA concentrations, and that maximum PhMA concentrations were well below the ACGIH BEI of 25 μg/g creatinine, it’s unlikely that benzene exposure was solely responsible for the elevated MUCA concentrations reported here. The median PhMA (a more reliable biomarker for benzene exposure) concentrations observed in this study were also lower than those measured at 3 h and 6 h post-fire for firefighters involved in training fires (Mayer et al., 2023).

Firefighters responding to WUI fires were also exposed to several other VOCs previously documented in structural firefighters during training fires, though the exposures reported here were generally lower (Fent et al., 2022; Mayer et al., 2023). These included biomarkers for exposure to acrolein (2CoEMA), styrene/ethylbenzene (MADA), xylenes (2MHA), acrylonitrile (2CyEMA), crotonaldehyde (3HMPMA), and 1, 3-butadiene (34HBMA, (E)-4HBeMA/(Z)-4HBeMA). Significant increases in metabolites of acrylamide (2CaEMA) and isoprene (4HMBeMA) were also observed, two VOCs that had not previously been reported to significantly increase during structural fire responses.

We also found that firefighters assigned to tasks associated with wildland firefighting (i.e. progressive hose lay, mop up, cutting line) had significantly higher changes in 2MHA concentrations compared to firefighters assigned to structure defense. Additionally, hand crew firefighters had significantly higher changes in 2MHA and 2CaEMA concentrations compared to the other ranks. It’s important to note that our sample size for battalion chiefs was low (n = 3), so caution should be exercised when interpreting these findings. However, VOCs are released during the combustion of both organic and synthetic materials, so it’s unsurprising that firefighters responding to WUI fires are exposed to these compounds.

Post WUI fire geometric mean concentrations of DPhP, a non-specific metabolite of TPhP, were higher than previously measured post-fire concentrations for firefighters participating in training fires involving modern home furnishings (Mayer et al., 2021). This finding is significant because OPEs like TPhP are present in synthetic materials and not the natural environment. Specifically, TPhP is added to products found in homes like nail polish, polyvinyl chloride plastic, polyurethane foam, and upholstered furniture (Gill et al., 2024; Mendelsohn et al., 2016). Though the majority of the firefighters with the highest concentrations of DPhP were assigned to structure defense, two of the top nine DPhP post-fire concentrations were collected from firefighters assigned to other tasks like cutting line/mop up and progressive hose lay. The half-life of DPhP is 9.6 days (Wang, X., et al., 2020), so it is possible these concentrations may reflect contributions from previous occupational exposures. However, given the significant elevation of DPhP post-fire concentrations relative to the concentrations at enrollment, some contribution from the WUI fire environment to the body burden is likely.

Although concentrations of BDCPP, a metabolite of TDCPP, did not increase from enrollment to post-fire in this study, the median concentrations at both enrollment (1.20 μg/g creatinine) and post-fire (1.17 μg/g creatinine) were higher than concentrations reported for firefighters and other first responders one month after responding to the 2023 Maui Wildfires (NIOSH, 2024). However, the geometric mean concentrations (1.26 μg/g creatinine at enrollment; 1.2 μg/g creatinine post-fire) remained below the geometric mean pre-fire concentrations (GM = 2.38 μg/g creatinine) noted by Mayer et al. (2021) in a study of firefighters responding to controlled residential fires. TDCPP has been associated with adverse health effects, which ultimately led to its usage restriction in certain products (Wang, C., et al., 2020). The observed lower concentrations of BDCPP may be a result of manufacturers replacing TDCPP with alternative OPEs (Hoehn et al., 2024). It’s also important to note that the urinary half-life for the metabolite of TDCPP (BDCPP) is 53.8 days (Wang, X., et al., 2020). So, it is possible the concentrations reported here may reflect other occupational or non-occupational sources unrelated to WUI fire responses. Regardless, exposures to OPEs like TPhP and TDCPP are certainly possible during WUI incidents due to the presence of synthetic materials in the fires.

Of the metal(loid)s evaluated in this study, antimony and cadmium were the only elements that significantly increased from enrollment to post-fire. Although cadmium concentrations rose modestly, all measured levels remained well below the ACGIH BEI of 5 μg/g creatinine (ACGIH, 2025). A recent assessment of individuals who responded to the 2023 Maui wildfires examined exposure to total arsenic, nickel, and chromium approximately one month after the fires. In the current study, total arsenic concentrations were significantly lower than those recorded during the Maui evaluation, with enrollment and post-fire median concentrations of 6.67 and 6.24 μg/g creatinine, respectively, compared to a median of 22.4 μg/g creatinine in the Maui study. Determining arsenic speciation in urine would be necessary to identify the specific forms present after the fire, since non-occupational sources, such as seafood consumption, can contribute to the arsenic levels observed in both evaluations. Notably, the Maui evaluation indicated that seafood intake likely played a role in the elevated arsenic levels reported (NIOSH, 2024). Nickel concentrations in the current study were also found to be lower than those measured in Maui, although there was a non-significant increase in median nickel concentrations from enrollment (0.73 μg/g creatinine) to post-fire (0.87 μg/g creatinine). Urinary chromium concentrations were comparable between the two evaluations; however, some firefighters in the current study had chromium concentrations exceeding the ACGIH BEI of 0.7 μg/L at both enrollment and post-fire. Because elevated concentrations were found both at enrollment and post-fire, it’s possible that the source of chromium was from other occupational or non-occupational sources, including supplements (Bethencourt-Barbuzano et al., 2025).

Additionally, a recent biomonitoring study of wildland firefighters reported significant increases in nickel, antimony, and cesium concentrations from pre-to post-wildfire response among non-smoking wildland firefighters (Paiva et al., 2024). Among the common metal(loid)s analyzed in both studies, antimony was the only one that showed a significant increase in both evaluations. However, geometric mean urinary antimony concentrations at both enrollment and post-fire remained below the NHANES 95th percentile, despite the statistically significant increase. A previous study also found antimony concentrations on structural firefighter’s skin, clothing, and PPE that was significantly elevated (Keir et al., 2020). In the future, firefighters’ exposures to metal (loid)s during WUI fires may increase as the use of lithium-ion batteries (LIBs), including those found in electric vehicles, becomes more widespread. Continued research evaluating the contribution of metal(loid)s to firefighters’ overall exposure burden will be important for advancing understanding in this area.

The health consequences of firefighters’ exposures during WUI fires are not well understood. Chronic exposure to many of the chemicals we evaluated have been associated with harmful effects, including increased risk of some types of cancer. Notably, Goodrich et al. assessed epigenetic modifications in the same population as this study and found that WUI fire exposure affected microRNA expression along pathways that may increase the risk of prostate and other cancers (Goodrich et al., 2025). Biomonitoring of firefighters responding to the January 2025 Los Angeles area urban conflagrations, which started in the WUI environment, showed changes in serum proteins associated with nucleotide synthesis and repair, oxidative stress response, and energy metabolism; and alterations in protein pathways associated with changes in metabolism and oxidative stress, immune and inflammatory responses, cellular barrier integrity and trafficking, and growth/cancer signaling (Furlong et al., 2026). Wildland firefighters are also believed to be at increased risk of lung cancer and cardiovascular disease mortality (Navarro et al., 2019), although much less is known about long-term risks specific to the complex multiple exposures that firefighters can incur from WUI fires.

This study is strengthened by its repeated measures design and a relatively large sample size (particularly the 88 paired enrollment and post-WUI fire response specimens), which is noteworthy compared to other exposure assessments that often rely on smaller sample sizes and/or single measurements (Cherry et al., 2021; Mayer et al. 2023, 2025; Paiva et al., 2024). The analyses included comprehensive chemical panels, encompassing PAHs, VOCs, OPEs, and metal(loid)s. Additionally, the use of creatinine-corrected concentrations enhances the reliability of the findings by accounting for variations in urine dilution among participants.

This study is not without its limitations. The urinary concentrations reported here, both at enrollment and post-fire may reflect occupational (e.g., other recent fire responses) unrelated to the WUI fire responses included in this evaluation, particularly given the relatively long elimination half-lives of certain chemicals evaluated, such as DPhP and BDCPP, as well as some metal(loid)s. Post-fire sample collection was standardized as much as possible given the field conditions, but the differences in the timing of the post-fire collection (one to 3 h after their shift) could have influenced results, potentially leading to an underestimation of exposure for some compounds. Some urine samples did not receive the mercury preservative within 10 h of collection, potentially allowing mercury loss. These samples were stored frozen (≤−20 °C or on dry ice) until preservative was added during aliquoting up to 48 h later, resulting in one to two freeze–thaw cycles. Although freezing may have reduced mercury loss, the stability of mercury in unpreserved frozen urine is uncertain and is a limitation of this study. Non-firefighting factors, including dietary influences on markers such as MUCA and the use of cosmetic products like nail polish containing TPhP, could also contribute to concentrations reported here (Jia et al., 2024; Weaver et al., 2000). Creatinine correction was used to normalize for urinary dilution, consistent with prior firefighter biomonitoring studies (Mayer et al., 2021, 2023; Fent et al., 2022) and enabling comparison to NHANES reference values. However, creatinine normalization can be affected by dehydration and intense physical exertion, both common during WUI fire response. Sweating is a natural thermoregulatory response to heat stress, and firefighters responding to WUI fires experience substantial physiological demands; these demands, combined with thermal stress from the ambient environment, active or smoldering fire, and the use of PPE, can lead to excessive sweating and dehydration. The significant reduction in urinary iodine concentrations from enrollment to post-fire observed in this study may reflect iodine loss through sweat rather than a reduction in exposure (Mao et al., 2001). Furthermore, DPhP is a metabolite of other OPEs including isopropylphenyl diphenyl phosphate, t-butylphenyl diphenyl phosphate, and 2-ethylhexyl diphenyl phosphate (Nishimaki-Mogami et al., 1988; Phillips et al., 2020; Shen et al., 2019) which raises the possibility that the concentrations observed may not be solely indicative of TPhP exposure.

The urinary results here provide an integrative measure of exposure from all sources and routes. However, we lacked comprehensive data on the use of PPE during these fire responses and did not analyze air or hand-wipe samples, which limited our ability to evaluate specific routes of exposure. Given the nature of the fire responses involved, it is unlikely that the firefighters in this study consistently wore respiratory protection, suggesting that both dermal and inhalation exposures contributed to the internal doses reported. We also did not evaluate all contaminants to which firefighters may be occupationally exposed, such as per- and polyfluoroalkyl substances (PFAS). We also did not have complete information on incident responses, so our analyses on the effect of rank and job assignment are somewhat limited. The sample size for some of our subgroups for the rank analysis were also small, which limited our statistical power. Finally, these findings are specific to WUI fire responses in California and may not be generalizable to WUI incidents in other regions or countries.

## 5. Conclusion

This study found compelling evidence that firefighters responding to WUI fires in California are exposed to a range of hazardous chemicals, including PAHs, VOCs, and select OPEs and metal(loid)s, with significant increases in urinary concentrations from enrollment to post-fire for several biomarkers. This study highlights that certain chemical exposures experienced by firefighters in WUI environments are comparable to those encountered during structural fire responses. In some cases, the maximum concentrations reported here were above applicable BEIs (i.e., 1-PYR, MUCA). Additionally, job assignment and rank influenced the degree of exposure to specific compounds.

Future research assessing firefighter exposures during WUI fires to other chemicals commonly found in the built environment, such as polybrominated diphenyl ethers and PFAS, could add substantial understanding to the existing body of research. Overall, this study emphasizes the need to research and develop best practices for PPE usage and decontamination practices during and after WUI responses. Given the concerns about cancer risk in this population, long-term exposure and health studies are needed. By building on this research and evaluating both exposure and effect biomarkers, along with PPE usage, researchers may identify strategies to reduce exposures during WUI fire response.

## Supplementary Material

Supplemental materials

## Figures and Tables

**Table 1:** Participant demographics^a^ and work characteristics.

		N (%)	Mean (SD)
Total participants		89^c^	
Age			37.9 (9.83)
Ethnicity	Hispanic	31 (35)	
	Non-Hispanic	58 (65)	
Race^b^ (n = 86)	White	68 (79)	
	All other races	24 (28)	
	Not reported	3	
Past military service	Yes	10 (11)	
	No	79 (89)	
Fire department	FD A	74 (83)	
	FD B	15 (17)	
Rank	Firefighter	38 (43)	
	Driver Operator	17 (19)	
	Captain	19 (21)	
	Hand Crew	12 (14)	
	Battalion Chief	3 (3)	
Years as a firefighter	<5	31 (35)	
	5 to <10	12 (13)	
	10 to <20	24 (27)	
	≥20	22 (25)	

a Sex of participants is not reported because fewer than 5 participants identified as female.

b Participants could select more than one race.

c One participant did not provide a urine sample at enrollment.

**Table 2:** Urine sample collected and fire response characteristics for subset of fire responses.

		N (%)
Full Study		
Participants with urine samples		89 (100)
Urine samples	Enrollment	88 (100)
	Fire response	125 (100)
	One WUI response	89 (71)
	Two to four WUI responses	36 (29)
Subset with Fire Response Details		
Participants		74 (83)
Urine samples	Total samples after fire responses	103 (100)
	One WUI response	74 (72)
	Two to four WUI responses	29 (28)
Primary Assignment	Other (e.g., cutting line, mop up, progressive hose lay)	30 (29)
	Structure defense	73 (71)
Fuels (n = 101)	Light to medium brush	10 (10)
	Light to medium brush & agricultural land	15 (15)
	Light to medium brush & structures	64 (63)
	Medium to heavy brush & structures	12 (12)
	Missing	2

**Table 3:** Polycyclic aromatic hydrocarbon (PAH) biomarker results (μg/g creatinine) for firefighters responding to wildland urban interface (WUI) fires.

Biomarker (Parent Chemical)	Collection Period	N (N of non-detects^f^)	Geometric Mean (GSD)	Median	Min-Max	P-value^d^	General Population 95% Percentile (Non-Smoker/Smoker^e^)
1-NAP (Naphthalene)	Enrollment	85^a^ (0)	0.95 (2.20)	0.83	0.27 – 9.48	<0.001	9.85/31.9
	Post-fire	122^bc^ (0)	4.35 (3.00)	4.39	0.44 – 50.53		
2-NAP (Naphthalene)	Enrollment	88 (0)	8.22 (1.83)	8.87	1.70 – 34.21	<0.001	19.7/30.5
	Post-fire	124^bd^ (0)	12.01 (2.02)	12.10	2.10 – 104.23		
2-FLU (Fluorene)	Enrollment	88 (0)	0.14 (1.75)	0.12	0.05 – 0.7	<0.001	0.695/2.35
	Post-fire	124^bd^ (0)	0.40 (2.36)	0.39	0.07 – 3.76		
3-FLU (Fluorene)	Enrollment	88 (2)	0.07 (2.34)	0.06	0.01 – 0.74	<0.001	0.331/1.73
	Post-fire	124^bd^ (0)	0.24 (2.43)	0.22	0.03 – 1.46		
1-PHE (Phenanthrene)	Enrollment	88 (0)	0.09 (1.82)	0.08	0.03 – 0.58	<0.001	0.307/0.569
	Post-fire	124^bd^ (0)	0.23 (2.56)	0.21	0.05- 2.72		
2-3-PHE (Phenanthrene)	Enrollment	88 (0)	0.11 (1.83)	0.10	0.05 – 1	<0.001	0.374/0.781
	Post-fire	124^bd^ (0)	0.37 (2.84)	0.32	0.05 – 5.61		
1-PYR (Pyrene)	Enrollment	88 (21)	0.09 (1.97)	0.08	< LOD–0.67	<0.001	0.420/0.914
	Post-fire	124^bd^ (7)	0.29 (2.72)	0.30	< LOD – 3.85		

g. Limit of detection for each metabolite: 1-NAP = 0.06 μg/L, 2-NAP = 0.09 μg/L, 2-FLU = 0.008 μg/L, 3-FLU = 0.008 μg/L, 1-PHE = 0.009 μg/L, 2–3-PHE = 0.01 μg/L, and 1-PYR = 0.07 μg/L.

h. Minimum detectable concentration based on the median creatinine concentrations: 1-NAP = 0.04 μg/g creatinine, 2-NAP = 0.06 μg/g creatinine, 2-FLU = 0.005 μg/g creatinine, 3-FLU = 0.005 μg/g creatinine, 1-PHE = 0.005 μg/g creatinine, 2–3-PHE = 0.006 μg/g creatinine, and 1-PYR = 0.04 μg/g creatinine.

a An interfering substance was present in three samples. Those sample were removed from analyses.

b One sample could not be linked with the study ID for all PAH metabolites. That sample was removed from analyses.

c An interfering substance was present in three samples, and one sample vial was empty when received by the lab for analysis.

d One sample vial was empty when received by the lab for analysis. That sample was removed from analyses.

e A random effects model was used to evaluate differences between enrollment and post-fire median concentrations.

f The 95th percentiles are for nonsmokers and smokers aged 18-49 years from the 2015-2016 NHANES cycle https://www.cdc.gov/exposurereport/report/pdf/Polycyclic%20Aromatic%20Hydrocarbon%20Metabolites%20FDA-p.pdf.

**Table 4:** Volatile organic compound (VOC) biomarker results (μg/g creatinine) for firefighters responding to wildland urban interface (WUI) fires.

Biomarker (Parent Chemical)	Collection Period	N (N of non-detects^d^)	Geometric mean (GSD)	Median	Min-Max	P-value^b^	General Population 95% Percentile (Non-Smoker/Smoker)^c^
2MHA (Xylene)	Enrollment	88 (5)	21.31 (2.21)	22.27	< LOD – 108.61	0.005	170/268
	Post-fire	124^a^ (6)	28.28 (2.74)	25.38	< LOD – 642.88		
2CaEMA (Acrylamide)	Enrollment	88 (0)	41.00 (1.50)	40.30	16.08 – 129.73	0.012	156/379
	Post-fire	124^a^ (0)	46.79 (1.51)	44.64	19.19 – 152.43		
1PMA (1-Bromopropane)	Enrollment	88 (10)	6.87 (3.43)	6.92	< LOD - 432.35	0.019	42.8/32.3
	Post-fire	124^a^ (12)	4.92 (3.04)	4.91	< LOD - 75.22		
2CoEMA (Acrolein)	Enrollment	88 (1)	61.31 (1.75)	63.05	< LOD - 257.80	<0.001	213/554
	Post-fire	124^a^ (0)	84.83 (1.75)	83.72	20.67-381.11		
2CyEMA (Acrylonitrile)	Enrollment	88 (15)	1.22 (2.94)	0.97	< LOD – 48.20	<0.001	58.6/422
	Post-fire	124^a^ (3)	8.27 (3.88)	8.69	< LOD – 81.14		
34HBMA (1,3-Butadiene)	Enrollment	88 (0)	249.12 (1.33)	245.62	124.60 – 570.55	0.006	510/721
	Post-fire	124^a^ (0)	277.03 (1.35)	279.89	101.50 – 675.61		
3HMPMA (Crotonaldehyde)	Enrollment	88 (0)	157.25 (1.56)	150.04	69.00 – 1091.44	<0.001	604/3600
	Post-fire	124^a^ (0)	201.45 (1.68)	199.28	62.23 – 927.71		
4HMBeMA (Isoprene)	Enrollment	88 (14)	2.37 (1.94)	2.45	< LOD – 11.06	<0.001	10.9/125
	Post-fire	124^a^ (6)	6.00 (2.61)	5.96	< LOD – 73.66		
MADA (Styrene, ethylbenzene)	Enrollment	88 (1)	123.33 (1.65)	124.03	< LOD – 440.71	0.019	267/538
	Post-fire	124^a^ (1)	142.81 (1.51)	145.90	< LOD – 435.36		
(E)-4HBeMA/(Z)-4HBeMA (1,3-Butadiene)	Enrollment	88 (4)	3.80 (2.02)	4.09	< LOD – 22.80	<0.001	12.2/72.7
	Post-fire	124^a^ (1)	5.60 (1.98)	5.13	< LOD – 45.39		
MUCA (Benzene)	Enrollment	88 (5)	49.10 (2.71)	41.77	< LOD – 2256.04	<0.001	N/A
	Post-fire	124^a^ (2)	97.42 (2.71)	90.15	< LOD – 1028.05		
PhMA (Benzene)	Enrollment	88 (37)	0.13 (1.52)	0.11	< LOD – 0.53	<0.001	N/A
	Post-fire	124^a^ (24)	0.22 (1.94)	0.20	< LOD – 1.14		

e. Minimum detectable concentration based on the median creatinine concentrations: 2MHA = 3.14 μg/g creatinine, 2CaEMA = 1.38 μg/g creatinine, 1PMA = 0.75 μg/g creatinine, 2CoEMA = 4.38 μg/g creatinine, 2CyEMA = 0.31 μg/g creatinine, 34HBMA = 3.30 μg/g creatinine, 3HMPMA = 1.07 μg/g creatinine, 4HMBeMA = 1.20 μg/g creatinine, MADA = 7.55 μg/g creatinine, (E)-4HBeMA/(Z)-4HBeMA = 0.38 μg/g creatinine, MUCA = 6.17 μg/g creatinine, PhMA = 0.09 μg/g creatinine.

a One sample could not be linked with the study ID for all VOC metabolites. One sample vial was empty when received by the lab for analysis. Those samples were removed from analyses.

b A random effects model was used to evaluate differences between enrollment and post-fire median concentrations.

c The 95th percentile was for people aged 18-49 years for nonsmokers and smokers from the 2015 to 2016 NHANES cycle https://www.cdc.gov/exposurereport/report/pdf/Volatile%20Organic%20Compound%20(VOC)%20Metabolites%20FDA-p.pdf.

d Limit of detection for each metabolite: 2MHA = 5.00 μg/L, 2CaEMA = 2.20 μg/L, 1PMA = 1.20 μg/L, 2CoEMA = 6.96 μg/L, 2CyEMA = 0.500 μg/L, 34HBMA = 5.25 μg/L, 3HMPMA = 1.70 μg/L, 4HMBeMA = 1.20 μg/L, MADA = 12.0 μg/L, (E)-4HBeMA/(Z)-4HBeMA = 0.600 μg/L, MUCA = 9.81 μg/L, PhMA = 0.15 μg/L.

**Table 5:** Organophosphate ester flame retardant (OPEs) biomarker results (μg/g creatinine) for firefighters responding to wildland urban interface (WUI) fires.

Biomarker (Parent Chemical)	Collection Period	N (N of non-detects^d^)	Geometric Mean (GSD)	Median	Min-Max	P-value^b^	General Population 95% Percentile^c^
DPhP (TPhP and EH-DPhP)	Enrollment	88 (1)	1.00 (2.39)	0.83	< LOD – 16.92	<0.001	4.08
	Post	123^a^ (1)	2.04 (2.63)	1.79	< LOD – 21.03		

a One sample could not be linked with the study ID for all OPE metabolites. An interfering substance was present in one sample. One sample vial was empty when received by the lab for analysis. Those samples were removed from analyses.

b A random effects model was used to evaluate differences between enrollment and post-fire median concentrations.

c The NHANES 95th percentile was for people aged 20 years and older from the 2017 to 2018 NHANES cycle https://www.cdc.gov/exposurereport/report/pdf/cgroup52_SSDPHP_2011-p.pdf.

d Limit of detection for DPhP = 0.10 μg/L; minimum detectable concentration based on median creatinine concentrations: 0.06 μg/g creatinine.

**Table 6:** Urine metal(loid)s results (μg/g creatinine) for firefighters responding to wildland urban interface (WUI) fires.

Biomarker	Collection Period	N (N of non-detects)	Geometric Mean (GSD)	Median	Min-Max	P-value^d^	General Population 95% Percentile (Non-Smoker/Smoker)^e^
Barium	Enrollment	88 (0)	1.32 (2.06)	1.49	0.23 – 5.63	<0.001	4.82/6.17
	Post	124^ab^ (1)	0.94 (2.18)	0.99	< LOD – 6.08		
Cadmium	Enrollment	88 (18)	0.08 (1.70)	0.08	< LOD – 0.28	0.042	0.41/0.67
	Post	124^ab^ (15)	0.09 (1.78)	0.09	< LOD – 0.50		
Iodine	Enrollment	86^c^ (0)	86.68 (1.94)	75.41	28.66 – 1337	<0.001	NA
	Post	125^a^ (0)	60.91 (1.68)	57.82	8.84 – 327.25		
Antimony	Enrollment	88 (7)	0.044 (1.78)	0.043	< LOD – 0.15	*<*0.001	0.14/0.19
	Post	124^ab^ (1)	0.096 (2.06)	0.081	< LOD – 0.75		
Thallium	Enrollment	88 (2)	0.15 (1.78)	0.15	< LOD – 0.64	0.001	0.44/0.36
	Post	124^ab^ (1)	0.13 (1.53)	0.13	< LOD – 0.42		

f. Limit of detection for each metal(loid): barium = 0.084 μg/L, cadmium = 0.055 μg/L, iodine = 2.4 μg/L, antimony = 0.18 μg/L, thallium = 0.017 μg/L.

g. Minimum detectable concentration based on the median creatinine concentrations: barium = 0.053 μg/g creatinine, cadmium = 0.035 μg/g creatinine, iodine = 1.51 μg/g creatinine, antimony = 0.11 μg/g creatinine, thallium = 0.011 μg/g creatinine.

a One sample could not be linked with the study ID for all metal(loid)s. That sample was removed from analyses.

b One sample vial was empty when received by the lab for analysis. That sample was removed from analyses.

c Two sample vials were not received by the lab. Those samples were not included in the analyses.

d A random effects model was used to evaluate differences between enrollment and post-fire median concentrations.

e The NHANES 95th percentile was for people aged 18-49 years for nonsmokers and smokers from the 2015 to 2016 NHANES cycle https://www.cdc.gov/exposurereport/report/pdf/Metals%20and%20Metalloids%20FDA-p.pdf.

## Appendix A. Supplementary data

Supplementary data to this article can be found online at https://doi.org/10.1016/j.ijheh.2026.114877.
