# Supplementary material for: Urinary biomarkers of PAHs, VOCs, OPEs, and metal(loid)s in firefighters responding in the wildland urban interface (WUI)
Source: Int J Hyg Environ Health. Author manuscript; Available in PMC 2026 Aug 19. (PMC13483172; doi:10.1016/j.ijheh.2026.114877)
Supplement: Supplementary file 1 [file NIHMS2201327-supplement-Supplemental_materials.docx]

Title: Urinary Biomarkers of PAHs, VOCs, OPEs, and Metal(loid)s in Firefighters Responding in the Wildland Urban Interface (WUI)

Authors and Affiliations

Miriam M. Calkins^a,k^, Alexander C. Mayer^a^, Derek J. Urwin^b,c,d^, Steve Bertke^a^, Christine Toennis^a^, Shawn C. Beitel^e^, Jeff Hughes^f^, Jamie Gabriel^b^, Antonia M. Calafat^g^, Christopher M. Reese^g^, Julianne Cook Botelho^g^, Maria Ospina^g^, Cynthia D. Ward-Momon^g^, Jeffery M. Jarrett^g^, Alberto J. Caban-Martinez^h^, Judith M. Graber^i^, Deborah Sammons^a^, Kathleen M. DuBose^j^, Jefferey L. Burgess^e^

^a^National Institute of Occupational Safety and Health, Centers for Disease Control and Prevention, Cincinnati, OH

^b^Los Angeles County Fire Department

^c^Department of Chemistry & Biochemistry, University of California Los Angeles, Los Angeles, CA

^d^International Association of Fire Fighters, Washington, D.C.

^e^Zuckerman College of Public Health, University of Arizona, Tucson, AZ

^f^Orange County Fire Authority

^g^Division of Laboratory Sciences, National Center for Environmental Health, Centers for Disease Control and Prevention, Atlanta, GA

^h^Leonard M. Miller School of Medicine, University of Miami, Miami, FL

^i^Rutgers, the State University of New Jersey, Piscataway, MJ

^j^Office of Wildland Fire, Department of the Interior

^k^Duke University, Division of Environmental Natural Sciences, Durham, NC

Corresponding Author: (Alexander C. Mayer)

Target Journal: International Journal of Hygiene and Environmental Health (IJHEH)

| **Supplemental Table 1:** Urinary Biomarkers and associated parent chemicals | | |
| --- | --- | --- |
| **Acronym** | **Analyte** | **Parent Chemicals** |
| **Polycyclic Aromatic Hydrocarbons** | | |
| 1-NAP | 1-hydroxynaphthalene | Naphthalene |
| 2-NAP | 2-hydroxynaphthalene | Naphthalene |
| 2-FLU | 2-hydroxyfluorene | Fluorene |
| 3-FLU | 3-hydroxyfluorene | Fluorene |
| 1-PHE | 1-hydroxyphenanthrene | Phenanthrene |
| 2-3PHE | 2-3-hydroxyphenanthrene | Phenanthrene |
| 1-PYR | 1-hydroxypyrene | Pyrene |
| **Volatile Organic Compounds** | | |
| (E)-4HBeMA, (Z)-4HBeMA | N-Acetyl-S-(4-hydroxy-2-buten-1-yl)-L-cysteine | 1,3-Butadiene |
| 34HBMA | N-Acetyl-S-(3,4-dihydroxybutyl)-L-cysteine | 1,3-Butadiene |
| 1PMA | N-Acetyl-S-(n-propyl)-L-cysteine | 1-Bromopropane |
| 3HPMA | N-Acetyl-S-(3-hydroxypropyl)-L-cysteine | Acrolein |
| 2CoEMA | N-Acetyl-S-(2-carboxyethyl)-L-cysteine | Acrolein |
| 2CaEMA | N-Acetyl-S-(2-carbamoylethyl)-L-cysteine | Acrylamide |
| 2CaHEMA | N-Acetyl-S-(2-carbamoyl-2-hydroxyethyl)-L-cysteine | Acrylamide |
| 2CyEMA | N-Acetyl-S-(2-cyanoethyl)-L-cysteine | Acrylonitrile |
| 1CyHEMA | N-Acetyl-S-(1-cyano-2-hydroxyethyl)-L-cysteine | Acrylonitrile |
| 2HEMA | N-Acetyl-S-(2-hydroxyethyl)-L-cysteine | Acrylonitrile, Vinyl chloride, ethylene oxide |
| MUCA | trans, trans-Muconic acid | Benzene |
| PhMA | N-Acetyl-S-(phenyl)-L-cysteine | Benzene |
| TTCA | 2-Thioxothiazolidine-4-carboxylic acid | Carbon disulfide |
| 3HMPMA | N-Acetyl-S-(3-hydroxypropyl-1-methyl)-L-cysteine | Crotonaldehyde |
| 2ATCA | 2-Aminothiazoline-4-carboxylic acid | Cyanide |
| MCaMA | N-Acetyl-S-(N-methylcarbamoyl)-L-cysteine | N, N-Dimethylformamide, Methyl isocyanate |
| PhGA | Phenylglyoxylic acid | Ethylbenzene, styrene |
| 4HMBeMA | N-Acetyl-S-(4-hydroxy-2-methyl-2-buten-1-yl)-L-cysteine | Isoprene |
| 2HPMA | N-Acetyl-S- (2-hydroxypropyl)-L-cysteine | Propylene oxide |
| MADA | Mandelic acid | Styrene, ethylbenzene |
| 2HPhEMA | N-Acetyl-S-(1-phenyl-2-hydroxyethyl)-L-cysteine  +  N-Acetyl-S-(2-phenyl-2-hydroxyethyl)-L-cysteine | Styrene |
| BzMA | N-Acetyl-S-(benzyl)-L-cysteine | Toluene, benzyl alcohol |
| 3MHA+4MHA | 3-Methylhippuric acid + 4-Methylhippuric acid | Xylene |
| 2MHA | 2-Methylhippuric acid | Xylene |
| **Organophosphate Esters** | | |
| DPhP | Diphenyl phosphate | Triphenyl phosphate ( TPhP),  Isopropylphenyl diphenyl phosphate  t-Butylphenyl diphenyl phosphate  2-Ethylhexyl diphenyl phosphate |
| BDCIPP | Bis(1,3-dichloro-2-propyl) phosphate | Tris(1,3-dichloro-2-propyl) phosphate (TDCPP) |
| DCP | Dicresyl phosphate | Tricresyl phosphates |
| BCIPP | Bis(1-chloro-2-propyl) phosphate | Tris(1-chloro-2-propyl) phosphate (TCPP) |
| DBuP | Dibutyl phosphate | Tributyl phosphate (TBuP) |
| DBzP | Dibenzyl phosphate | Tribenzyl phosphate |
| BCEP | Bis(2-chloroethyl) phosphate | Tris(2-chloroethyl) phosphate (TCEP) |
| iPPPP | 2-((isopropyl)phenyl)phenyl hydrogen  phosphate | Isopropylphenyl diphenyl  phosphate |
| tBPPP | 4-((tert-butyl)phenyl) phenyl hydrogen  phosphate | Tertbutylphenyl diphenyl phosphate |
| **Other Biomarkers** | | |
| TBBA | 2,3,4,5-Tetrabromobenzoic acid | 2-Ethylhexyl 2,3,4,5-tetrabromobenzoate (TBB) |

| **Supplemental Table 2.** Volatile organic compound (VOC) biomarker results (µg/g creatinine) for firefighters responding to wildland urban interface (WUI) fires. | | | | | |
| --- | --- | --- | --- | --- | --- |
| Biomarker (Parent Chemical) | Collection Period | N (N of non-detects^b^) | Geometric mean (GSD) | Median | Min-Max |
| 3MHA+4MHA  (Xylenes) | Enrollment | 88 (1) | 111.08 (2.00) | 104.29 | < LOD – 967.07 |
|  | Post | 124^a^ (1) | 130.34 (2.13) | 119.03 | < LOD – 1,439 |
| MCaMA  (N,N-Dimethylformamide; Methyl isocyanate) | Enrollment | 88 (0) | 87.69 (1.88) | 88.67 | 11.32 – 264.12 |
|  | Post | 124^a^ (0) | 88.24 (1.95) | 97.47 | 4.44 – 370.63 |
| 2ATCA  (Cyanide) | Enrollment | 88 (12) | 74.28 (2.77) | 63.44 | < LOD – 1,312.53 |
|  | Post | 124^a^ (11) | 67.98 (2.28) | 65.20 | < LOD – 716.31 |
| BzMA  (Toluene, benzyl alcohol) | Enrollment | 88 (0) | 4.96 (1.89) | 4.44 | 1.664 – 30.83 |
|  | Post | 124^a^ (0) | 5.13 (1.94) | 4.91 | 1.864 - 93.69 |
| 1CyHEMA  (Acrylonitrile) | Enrollment | 88 (85) | NC | NC | < LOD – 5.75 |
|  | Post | 124^a^ (69) | NC | NC | < LOD – 17.05 |
| 2CaHEMA  (Acrylamide) | Enrollment | 88 (57) | NC | NC | < LOD – 11.62 |
|  | Post | 124^a^ (60) | NC | NC | < LOD – 19.39 |
| 2HEMA (Acrylonitrile, Vinyl chloride, ethylene oxide) | Enrollment | 88 (67) | NC | NC | < LOD – 1.89 |
|  | Post | 124^a^ (79) | NC | NC | < LOD – 1.80 |
| 2HPMA  (Propylene oxide) | Enrollment | 88 (4) | 24.34 (2.12) | 23.94 | < LOD – 449.22 |
|  | Post | 124^a^ (1) | 30.27 (2.47) | 26.17 | < LOD – 1,708.33 |
| 3HPMA  (Acrolein) | Enrollment | 88 (0) | 196.52 (1.92) | 178.01 | 66 – 1,567.47 |
|  | Post | 124^a^ (0) | 210.80 (1.78) | 203.52 | 57.85 – 805.20 |
| 2HPhEMA  (Styrene) | Enrollment | 88 (58) | NC | NC | < LOD – 6.3 |
|  | Post | 124^a^ (54) | NC | NC | < LOD – 6.85 |
| PhGA  (Ethylbenzene, styrene) | Enrollment | 88 (16) | 53.76 (3.23) | 90.341 | 6.937 – 304.86 |
|  | Post | 124^a^ (28) | 52.77 (3.37) | 76.21 | < LOD – 286.99 |
| TTCA  (Carbon disulfide) | Enrollment | 88 (47) | NC | NC | < LOD – 504.89 |
|  | Post | 124^a^ (70) | NC | NC | < LOD – 315.39 |
| NC = Not calculated   a. One sample could not be linked with the study ID for all VOC metabolites. One sample vial was empty when received by the lab for analysis. Those samples were removed from analyses. b. Limit of detection for each metabolite: 3MHA+4MHA= 8.00 μg/L, MCaMA=6.26 μg/L, 2ATCA= 29.5 μg/L, BzMA=0.50 μg/L, 1CyHEMA= 2.60 μg/L, 2CaHEMA= 9.40 μg/L, 2HEMA=0.791 μg/L, 2HPMA=5.30 μg/L, 3HPMA=13.0 μg/L, 2HphEMA=1.00 μg/L, PhGA=12.0 μg/L, TTCA=11.2 μg/L. c. Minimum detectable concentration based on the median creatinine concentrations: 3MHA+4MHA= 5.03 μg/g creatinine, MCaMA=3.94 μg/g creatinine, 2ATCA= 18.6 μg/g creatinine, BzMA=0.31 μg/g creatinine, 1CyHEMA= 1.64 μg/g creatinine, 2CaHEMA= 5.91 μg/g creatinine, 2HEMA=0.50 μg/g creatinine, 2HPMA=3.33 μg/g creatinine, 3HPMA=8.18 μg/g creatinine, 2HphEMA=0.63 μg/g creatinine, PhGA=7.55 μg/g creatinine, TTCA=7.04 μg/g creatinine. | | | | | |

| **Supplemental Table 3.** Organophosphate ester flame retardant (OPEs) biomarker results^a^ (µg/g creatinine) for firefighters responding to wildland urban interface (WUI) fires | | | | | |
| --- | --- | --- | --- | --- | --- |
| Biomarker (Parent Chemical) | Collection Period | N (N of non-detects^e^) | Geometric Mean (GSD) | Median | Min-Max |
| BCEtP  (TCEP) | Enrollment | 88 (19) | 0.24 (2.81) | 0.22 | < LOD – 8.42 |
|  | Post | 124^b,c^ (14) | 0.29 (2.80) | 0.26 | < LOD – 8.71 |
| BCIPP (TCPP) | Enrollment | 87^d^ (27) | 0.14 (2.54) | 0.08 | < LOD – 5.99 |
|  | Post | 124^b,c^ (32) | 0.13 (2.61) | 0.094 | < LOD – 3.85 |
| BDCPP (TDCPP) | Enrollment | 88 (0) | 1.26 (2.26) | 1.20 | 0.12 – 19.16 |
|  | Post | 124^b,c^ (0) | 1.2 (1.96) | 1.17 | 0.21 – 7.92 |
| DBuP (TBuP) | Enrollment | 88 (61) | NC | NC | < LOD – 0.51 |
|  | Post | 124^b,c^ (64) | NC | NC | < LOD – 0.75 |
| TBBA (TBB) | Enrollment | 88 (79) | NC | NC | < LOD – 0.049 |
|  | Post | 123^b,e^ (116) | NC | NC | < LOD – 0.045 |
| iPPPP | Enrollment | 88 (77) | NC | NC | < LOD – 0.161 |
|  | Post | 124^b,c^ (73) | NC | NC | < LOD – 0.54 |
| tBPPP | Enrollment | 88 (76) | NC | NC | < LOD – 0.36 |
|  | Post | 124^b,c^ (101) | NC | NC | < LOD – 0.24 |
| NC= Not calculated   a. All samples for DBzP and DCP were below the limit of detection (LOD). b. One sample could not be linked with the study ID for all OPE metabolites c. One sample vial was empty when received by the lab for analysis. d. An interfering substance was present in one sample. e. An interfering substance was present in one sample and one sample vial was empty when received by the lab for analysis. f. Limit of detection for each metabolite: BCEtP=0.10 μg/L , BCIPP=0.10 μg/L, BDCPP=0.10 μg/L, BDuP=0.10 μg/L, DBzP=0.05 μg/L, DCP= 0.50 μg/L, TBBA=0.05 μg/L, iPPPP=0.05 μg/L, tbPPP=0.05 μg/L. g. Minimum detectable concentration based on the median creatinine concentrations: BCEtP=0.10 μg/g creatinine, BCIPP=0.06 μg/g creatinine, BDCPP=0.06 μg/g creatinine, BDuP=0.06 μg/g creatinine, DBzP=0.03 μg/g creatinine, DCP= 0.50 μg/g creatinine, TBBA=0.03 μg/g creatinine, iPPPP=0.03 μg/g creatinine, tbPPP=0.03 μg/g creatinine. | | | | | |
|  | | | | | |

| **Supplemental Table 4.** Urine metal(loid)s^a^ results (µg/g creatinine) for firefighters responding to wildland urban interface (WUI) fires. | | | | | |
| --- | --- | --- | --- | --- | --- |
| Biomarker | Collection Period | N (N of non-detects) | Geometric Mean (GSD) | Median | Min-Max |
| Chromium | Enrollment | 88 (57) | NC | NC | < LOD – 4.3 |
|  | Post-fire | 124^b,c^ (55) | NC | NC | < LOD – 4.62 |
| Cobalt | Enrollment | 88 (3) | 0.11 (1.73) | 0.11 | < LOD – 0.59 |
|  | Post-fire | 124^b,c^ (3) | 0.12 (1.65) | 0.11 | < LOD – 0.84 |
| Cesium | Enrollment | 87^c^ (0) | 4.12 (1.70) | 4.17 | < LOD – 15.5 |
|  | Post-fire | 124^b,c^ (0) | 4.08 (1.49) | 4 | 1.174 – 17.4 |
| Mercury | Enrollment | 86^d^ (26) | 0.19 (2.25) | 0.15 | < LOD – 1.27 |
|  | Post-fire | 125^b^ (32) | 0.19 (2.24) | 0.18 | < LOD – 1.64 |
| Manganese | Enrollment | 88 (54) | NC | NC | < LOD – 0.81 |
|  | Post-fire | 124^b,c^ (58) | NC | NC | < LOD – 0.65 |
| Molybdenum | Enrollment | 88 (0) | 35.7 (1.97) | 36.1 | 3.357 – 179 |
|  | Post-fire | 124^b,c^ (0) | 31.4 (1.78) | 34.2 | 7.853 – 125 |
| Nickel | Enrollment | 88 (17) | 0.67 (2.31) | 0.73 | < LOD – 6.73 |
|  | Post-fire | 123^b,f^ (15) | 0.79 (2.08) | 0.87 | < LOD – 3.58 |
| Lead | Enrollment | 88 (1) | 0.21 (1.66) | 0.21 | < LOD – 0.65 |
|  | Post-fire | 124^b,c^ (0) | 0.21 (1.51) | 0.21 | 0.069 – 0.76 |
| Tin | Enrollment | 88 (16) | 0.30 (2.06) | 0.25 | < LOD – 3.23 |
|  | Post-fire | 124^b,c^ (17) | 0.32 (2.23) | 0.27 | < LOD – 9.66 |
| Strontium | Enrollment | 87^c^ (0) | 98.9 (1.92) | 105 | < LOD – 434 |
|  | Post-fire | 123^b,e^ (0) | 85.5 (1.94) | 91.5 | < LOD – 416 |
| Arsenic | Enrollment | 88 (0) | 8.2 (2.42) | 6.67 | 2.153 – 89.9 |
|  | Post-fire | 123^b,f^ (0) | 7.62 (2.61) | 6.24 | < LOD – 100 |
| Tungsten | Enrollment | 88 (7) | 0.07 (2.72) | 0.08 | < LOD – 1 |
|  | Post-fire | 121^b,g^ (1) | 0.08 (2.52) | 0.06 | < LOD – 0.94 |
| Uranium | Enrollment | 88 (5) | 0.007 (2.52) | 0.006 | < LOD – 0.046 |
|  | Post-fire | 124^b,c^ (2) | 0.007 (1.80) | 0.007 | < LOD – 0.044 |
| NC= Not calculated   a. Over 90% of samples were below the limit of detection for beryllium and platinum, so no calculations were performed. b. One sample could not be linked with the study ID for all metal(loid)s. That sample was removed from analyses. c. One sample vial was received empty or had insufficient quantity for analysis. d. Two sample vials were not received by the lab. Those samples werenot included in the analysis. e. One sample vial was empty when received by the lab, and one sample vial has insufficient quantity for analysis. f. Two samples had insufficient quantity for analysis. g. One sample vial was empty when received by the lab, and three sample vials had insufficient quantity for analysis. h. Limit of detection for each metal(loid): beryllium= 0.02 μg/L, chromium=0.19 μg/L, cobalt=0.024 μg/L, cesium=0.13 μg/L, mercury=0.13 μg/L, manganese= 0.068 μg/L, molybdenum=0.45 μg/L, nickel=0.31 μg/L, platinum=0.0063 μg/L, lead= 0.022 μg/L, tin= 0.2 µg/L, arsenic= 0.23 μg/L, strontium=3.2 μg/L, tungsten= 0.015 μg/L, uranium=0.0024 μg/L i. Minimum detectable concentration based on the median creatinine concentrations: beryllium= 0.01 μg/g creatinine, chromium=0.12 μg/g creatinine, cobalt=0.015 μg/g creatinine, cesium=0.082 μg/g creatinine, mercury=0.082 μg/g creatinine, manganese= 0.043 μg/g creatinine, molybdenum=0.28 μg/g creatinine, nickel=0.19 μg/g creatinine, platinum=0.004 μg/g creatinine, lead= 0.014 μg/g creatinine, tin= 0.13 µg/g creatinine, arsenic= 0.14 μg/g, strontium=2.01 μg/g creatinine, tungsten= 0.009 μg/g creatinine, uranium=0.0015 μg/g creatinine. | | | | | |
